# Can Antiviral Drugs Contain Pandemic Influenza Transmission?

**DOI:** 10.1371/journal.pone.0017764

**Published:** 2011-03-28

**Authors:** Niels G. Becker, Dingcheng Wang

**Affiliations:** National Centre for Epidemiology and Population Health, The Australian National University, Canberra, Australian Capital Territory, Australia; Albert Einstein College of Medicine, United States of America

## Abstract

Antiviral drugs dispensed during the 2009 influenza pandemic generally failed to contain transmission. This poses the question of whether preparedness for a future pandemic should include plans to use antiviral drugs to mitigate transmission.

Simulations using a standard transmission model that allows for infected arrivals and delayed vaccination show that attempts to contain transmission require relatively few antiviral doses. In contrast, persistent use of antiviral drugs when the reproduction number remains above 1 use very many doses and are unlikely to reduce the eventual attack rate appreciably unless the stockpile is very large. A second model, in which the community has a household structure, shows that the effectiveness of a strategy of dispensing antiviral drugs to infected households decreases rapidly with time delays in dispensing the antivirals. Using characteristics of past pandemics it is estimated that at least 80% of primary household cases must present upon show of symptoms to have a chance of containing transmission by dispensing antiviral drugs to households. To determine data needs, household outbreaks were simulated with 50% receiving antiviral drugs early and 50% receiving antiviral drugs late. A test to compare the size of household outbreaks indicates that at least 100–200 household outbreaks need to be monitored to find evidence that antiviral drugs can mitigate transmission of the newly emerged virus.

Use of antiviral drugs in an early attempt to contain transmission should be part of preparedness plans for a future influenza pandemic. Data on the incidence of the first 350 cases and the eventual attack rates of the first 200 hundred household outbreaks should be used to estimate the initial reproduction number *R* and the effectiveness of antiviral drugs to mitigate transmission. Use of antiviral drugs to mitigate general transmission should cease if these estimates indicate that containment of transmission is unlikely.

## Introduction

The threat from avian influenza H1N5 prompted many countries to establish a stockpile of antiviral drugs, [1], [2], [3], [4], such as oseltamivir and zananivir. The size of the antiviral stockpile and its proposed use, therapy or prophylaxis, were keenly debated during the preparation of pandemic management plans. The emergence of pandemic H1N1 in 2009 prompted a variety of strategies for the use of antiviral drugs and motivates this look at the use of antiviral drugs for prophylaxis and implications for decisions on the size of an antiviral stockpile for a future pandemic.

The possibility of using antiviral drugs for prophylaxis, to mitigate transmission of pandemic influenza, arises because their use to protect against currently circulating strains of influenza indicates a reduced chance of being infected [5], [6], [7], [8], [9]. Also observed are reduced levels of virus shedding [5], [6], [10], [11], [12], [13], [14], which suggests a reduction in infectivity in the event of a breakthrough infection. Use of these observations in modeling studies suggests that stockpiles of antiviral drugs held by some nations are sufficiently large to defer the peak of the epidemic until a newly developed vaccine is available to control transmission [15], [16], [17], [18]. These results could be expected to apply to pandemic H1N1 since, with a reproduction number estimated to be of the order 1.2-1.5 in some localities [19], [20], its transmissibility is relatively modest.

In practice, the antiviral drugs dispensed during the 2009 influenza pandemic generally failed to contain transmission. This prompts us to ask why timely administration of antiviral drugs to a sufficient number of cases, exposed individuals and individuals at high risk of exposure did not occur. Could we have done better? On a future occasion, should we even attempt to contain transmission with the assistance of antiviral drugs? A consideration of these questions will inform preparedness plans for the next pandemic.

Some argue that using antiviral drugs to mitigate transmission merely wastes doses that are needed to treat cases experiencing severe disease. Here ‘dose’ means a course of antiviral drugs, typically lasting seven days. The fear of wastage is fed by the fact that the protective effect of antiviral drugs acts only for the duration of the dose (e.g. 7 days), so that individuals might need several doses during a pandemic. On the other hand, if prophylactic use of antiviral drugs is able to reduce the total number individuals infected then there will be fewer cases with severe disease in need of treatment with antiviral drugs. The optimal allocation of antiviral doses to treatment and prophylaxis depends on the size of the stockpile, effectiveness of antiviral drugs for treatment and protection from infection, as well as the transmission and disease progression characteristics of the new virus strain. Many of these factors will not be known prior to the pandemic. However, it is clearly worth asking whether a relatively modest number of antiviral doses used for prophylaxis might be able to reduce the eventual attack rate substantially.

Here we use simple models that contain the key features needed to assess the impact of using antiviral drugs to mitigate transmission. The aim is to clarify the potential benefit of timely use of antivirals for prophylaxis and its limitations. Specifically, we ask whether use of antiviral drugs should be included in an attempt to contain an emerged pandemic, we provide guidance on the size of stockpile needed for an attempt at containment and consider what data need to be collected at the start of a pandemic to assess the prophylactic effectiveness of antiviral drugs against the new virus strain.

## Methods

### The basic model

**Figure 1:**
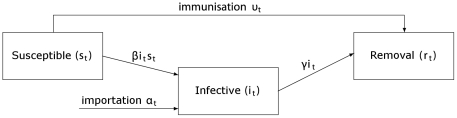
Baseline transmission model. Mass vaccination and arrival of infected individuals from other locations has been added to the standard SIR transmission model.

To assess the potential for antiviral drugs to mitigate transmission, we begin with the baseline model depicted in Figure 1, in which homogeneous individuals mix uniformly and experience transitions between the *Susceptible*, *Infective* and *Removal* states over time. A removal is an individual who is immune as a consequence of vaccination or recovery from an infection. Let 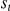, 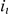 and 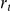 denote the proportion of individuals who are susceptible, infectious and removed at time 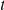. The equations describing transmission and recovery are
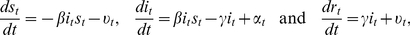
(1)where 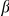 governs the rate of transmission and 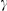 is the recovery rate. For our purpose, we have added to the standard SIR epidemic model a rate 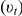 of immunisation by vaccination and a rate 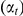 of importing newly infected individuals from other locations. These two rates may be time-dependent. The initial reproduction number is the number of secondary infections generated by a typical single primary case at the beginning when no one else has been infected. For this model it is given by 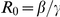. We do not refer to this 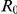 as the *basic* reproduction number because we allow for the possibility that awareness of a new pandemic strain may have changed behaviour and individuals may have some immunity against the new strain from previous exposure to other influenza strains.

Our concern is with a strain of influenza that is newly emerged and so individuals can be vaccinated only when a strain-specific vaccine has been developed and tested. To accommodate this delay, the rate of immunising susceptible individuals is assumed to have the form
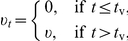
where 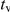 is the time when the new vaccine is ready to be dispensed. A time-varying rate of importing new infectives is realistic, but here we restrict attention to a constant importation rate 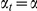.

Our main focus is on using antiviral drugs for prophylaxis, to hopefully contain or delay widespread transmission. For the moment suppose that each individual is symptomatic and presents to the health service following onset of their symptoms. We assume that each newly diagnosed case triggers the dispensing of 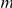 doses of antiviral drugs to individuals who have been exposed to or are a potential contact of that case. It is meaningful to allow non-integer values for 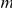 if we interpret it to be the average number of doses dispensed per case. In our model the effect of dispensing 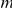 antiviral doses per case is to reduce the transmission rate 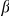 to 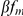, where the factor 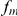 decreases as 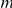 increases and 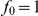. Here we use the form
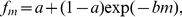
(2)for a variety of values of 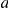 and 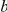 satisfying 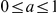 and 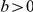, so that 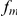 decreases from 1 to 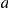 as 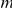 increases. This form for 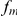 acknowledges that the first few doses dispensed are likely to reduce transmission more effectively because they target the closest associates of the case. The effect on the reproduction number is to reduce it from 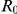 to 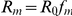, which is less than 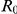 unless 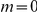.

To monitor the depletion of the stockpile of antiviral drugs we define 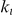  =  (total number of doses in the stockpile remaining at time 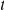)/(population size).

Then 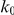, the initial number of doses per individual, is the initial size of the stockpile relative to the population size. When we dispense 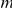 doses for each new case we find
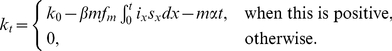
(3)


A variety of values for the parameters 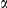, 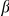, 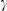, 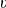, 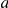 and 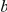 are used. In Table 1 we show baseline values for these parameters that seem relevant in planning preparedness for pandemic influenza, where the values of 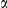, 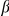, 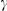 and 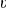 are rates per day. Results presented here are based on these baseline parameter values unless indicated otherwise. Initially we assume that everyone is susceptible, i.e. 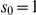 and 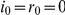. Transmission is seeded by the importation of infectives.

**Table 1:** Baseline values for model parameters.

*α*	*β*	*γ*	*R*_0_	*t*_v_	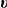	*a*	*b*
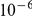	0.375	0.25	1.5	150	0.01	0.5	0.2

The recovery rate 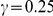 gives a mean infectious period of four days, the vaccination rate 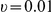 means that once developed the vaccine can be given to 1% of the population per day and 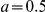 means that the chance of transmission per close contact can be reduced by at most 50% by liberal use of antiviral drugs. The value 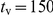 days assumes that it takes five months to develop a vaccine and get it ready for distribution.

A baseline initial value of 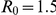, when planning to prepare for pandemic influenza, seems sensible on the basis of past experience. Attack rates observed during the pandemics of 1918, 1957, 1968 and 2009 are, for the most part, consistent with 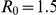, or less. It is, of course, possible that a pandemic strain with a higher transmission rate might evolve. Indeed, the basic reproduction number of the 1918 pandemic strain was almost certainly substantially higher, but compliance with public health measures based on social distancing during this pandemic was high because of the recognised severity of the disease. This is evident by observing how incidence changed as social distancing measures were introduced and removed; see Caley *et al*. [21]. As a result, although susceptibility was uniformly high the effective reproduction number, prior to depletion of susceptibles, was about 1.5. A high level of compliance is also likely in the event of a future pandemic strain with severe disease, suggesting that an initial effective reproduction number below 1.5 is likely from social distancing measures alone.

### Distributing antiviral drugs to affected households

It is natural that individuals responsible for distributing antiviral drugs are concerned about wastage in attempts to reduce transmission, when these drugs are thought necessary to treat severe cases and to protect essential-service personnel, such as health care workers and police, [17]. Faced with competing demands it is tempting to limit community distribution of antiviral drugs to cases with laboratory-confirmed infection and individuals with confirmed exposure. Unfortunately, laboratory confirmation and contact tracing are time consuming and insistence on such confirmation makes it impossible to administer antiviral drugs quickly enough to contain transmission. A strategy of dispensing antiviral drugs quickly and liberally to household members as soon as the first household case presents seems to be what is needed. A focus on households helps to clarify who is targeted for antiviral prophylaxis and the co-location of its members makes timely dispensing to exposed individuals feasible. We therefore look at transmission in a community of households with a focus on timeliness and transmission characteristics that make containment of transmission feasible. For such a community is useful to work with the reproduction number for infected households, [22], [23], [24], which we denote 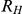.

To incorporate the effect of antiviral drugs on transmission into the calculation of 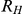 and the mean number of eventual cases, we adopt the effect formulation of Glass and Becker [25]. They model the effect of antiviral drugs by a change in the population dynamics of the virus population within the host and translate this to the corresponding change in 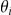, the probability that a susceptible individual avoids being infected by a single infected household member of generation 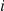. Infectives of one generation are the individuals infected by the infectives of the previous generation, where household generation 0 contains only the primary household case. For our purpose we also include the corresponding effect on 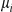, the mean number of cases a generation-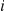 infective generates outside their household. As in [25], individuals who are not infectives of generations 0 and 1 are assumed to receive antiviral drugs before being infected and therefore derive the full protective effect of these drugs. Then the values of the probabilities 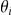 are same for 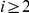. As in [25], we use a Reed-Frost model, [26], [27], for within-household transmission with the modification that the probability of avoiding infection is generation-dependent. The 2001 Australian census data was used to allocate a distribution to household size. With these specifications we compared the value of the reproduction number 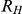 for three different settings, namely when (i) no antiviral drugs are dispensed, (ii) doses are dispensed to household members two days after the primary case is infected, and (iii) doses are dispensed four days after the primary case is infected.

In order to determine the largest fraction of non-compliance that still permits transmission to be contained, we also compute the effect of antiviral drugs on the reproduction number 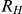 when a fraction 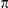 of primary household cases fails to present early enough for the household to receive antiviral drugs.

Finally, we determine how many household outbreaks might need to be observed to provide evidence that the antiviral drugs are indeed effective against the newly emerged virus strain. Here we look at establishing effectiveness via a simple comparison of the mean outbreak size in households that receive antiviral drugs early with the mean outbreak size in households that receive them late. This comparison of means must accommodate heterogeneity in variances and a number of tests permit this. We have chosen to use the Alexander-Govern test [28], because its computations are relatively simple, good performance has been demonstrated [29] and it provides a simple and direct way to combine the comparisons for households of different sizes.

## Results

### Transmission: contained or not contained

**Figure 2:**
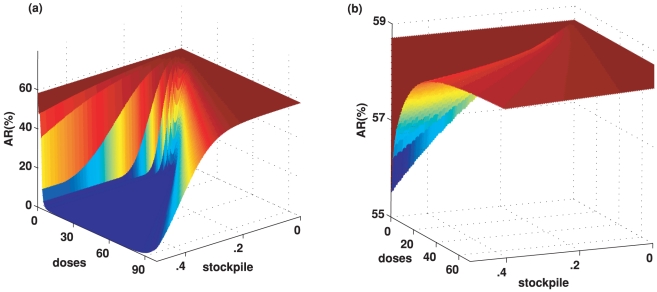
Eventual attack rate (*AR*). Percentage of the population infected, as predicted by the baseline model with (a) 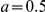 and (b) 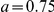, for different 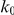 (stockpile size, as a proportion of the population size) and 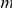 (number of antiviral doses dispensed per case). Colours on the graphs range from dark blue (low values) to dark red (high values).

The model given by equations (1)–(3) was used, with a range of plausible parameters values, to determine the eventual attack rate (percentage of the population infected). The consistent findings are illustrated in Figure 2, which shows the eventual attack rate as given by the model with (a) 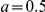 and (b) 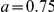 and other parameters assuming the baseline values of Table 1. The smallest value for 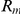 is 0.75 when 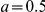 and 1.125 when 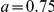.

For all parameter values, increasing 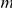 from zero decreases the transmission rate 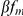 and this is reflected in a decline in the eventual attack rate 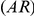. For larger values of 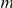, we see an increase in 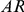 as 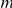 increases. This arises because the value of the transmission rate 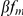 returns to 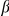 as the stockpile is depleted and epidemic transmission resumes (slightly tempered by a depletion of susceptibles).

When 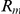 can be brought below 1, as in Figure 2(a), there is a very steep decline in 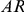 as 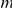 increases and 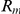 approaches 1. Note that 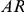 remains very low for a substantial range of values of 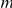. The range of 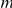 values for which transmission is contained depends on the size of the stockpile. The existence of a wide range of near-optimal values for 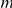 and the fact that transmission is contained for any value of 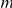 in this range provide realistic scope for practical and effective use of antiviral drugs for prophylaxis.

In contrast, when 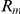 cannot be brought below 1, as in Figure 2(b), a small value of 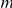 can induce a reduction in the attack rate. However, noting the scale on the vertical axis in Figure 2(b), we see that the reduction is small and very localised. It is difficult to utilise this optimal dosage in practice because its value depends on factors that are unknown and difficult to estimate with adequate precision.

### Number of doses of antiviral drugs dispensed

The eventual number of antiviral doses dispensed per community member can be computed numerically from equations (1)–(3). When transmission can be contained, it is more informative to work with a simple expression that approximates antiviral usage, because this expression reflects directly how various factors affect the usage.

When 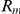 remains below 1 there is relatively little depletion of susceptibles, so we may write
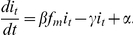
Then the fraction of infectives is soon near its equilibrium value of 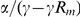 and the number of doses of antiviral drugs dispensed per community member is approximately
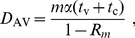
(4)where 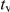 is the time when the vaccine is ready to be dispensed and 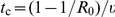 is the additional time required to reach a vaccination coverage that brings the effective reproduction number below 1. By way of illustration, note that equation (4) indicates that the required number of antiviral drug doses will be equal to 1.2% of the population size when 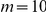 and other parameters assume their baseline value. This allows for one imported infective per day for every million population members, for a duration of six months, and all the transmission chains they generate. Prior to the 2009 pandemic many nations held antiviral stockpiles with doses that numbered more than 20% of their population. Only a small fraction of this would be needed for sustained containment of transmission until the vaccine controls transmission.

The 1.2% we calculated above is based on sustained containment for six months. When containment is not achievable we would become aware of this quite early and would abandon the attempt of containment having spent a small fraction of the stockpile. For example, Becker *et al.* [30] show that a useful estimate of the initial reproduction number is obtained once the cumulative incidence reaches 350–500 cases. With 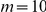 doses per case and a population of 1 million, we would therefore abandon the attempt at containment having spent antiviral doses numbering less than 0.05% of the population size if the estimate of 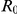 indicates containment is infeasible.

**Figure 3:**
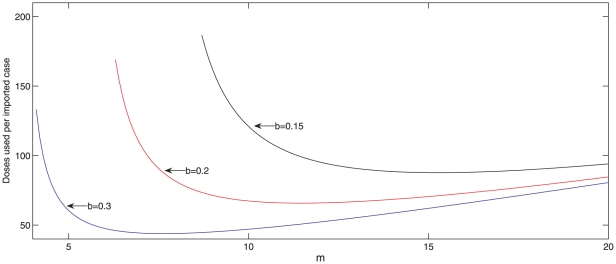
Doses used per imported case. Mean number of doses used to contain each outbreak initiated by one infected arrival, when 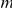 doses are dispensed for every case.

More generally, the total number of antiviral doses used, as given by (4), increases linearly with the rate of importations (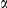), and the time until the vaccine is able to control transmission (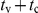). Together, these terms contribute the factor 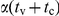, which is the mean number of importations of infected cases from the start of the pandemic until the vaccine is able to reduce the reproduction number below 1. The remaining factor in (3) is 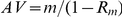, the mean number of doses dispensed for each outbreak initiated by a single infective. Its dependence on 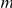 is shown in Figure 3. We see that 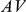 declines rapidly to a minimum as 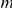 increases just beyond the value required to bring 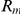 below 1. The optimal 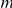 occurs when 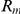 is about 0.8 and the gradual increase in 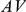 for larger values of 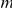 indicates that nothing is gained by striving to achieve a value of 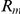 smaller than 0.8. Figure 3 illustrates that this conclusion is not sensitive to our assumed value of 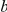.

The encouraging conclusion that a relatively small stockpile of antiviral drugs is needed for an attempt to contain the pandemic locally is not a consequence of the specific model (1). The same conclusion is reached from branching process models under quite general assumptions about characteristics of transmission and disease progression.

The enormous benefit of local containment of a pandemic virus strain can be realised only when (i) antiviral drugs are effective enough to reduce the reproduction number from its initial value of 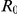 to a value below 1, and (ii) timely distribution of antiviral drugs to appropriate individuals is possible in practice. In retrospect, pandemic H1N1 influenza in 2009 seemed to satisfy condition (i), but condition (ii) was not realised. We now consider some possible reasons for the failure to distribute antiviral drugs effectively.

### Distributing antiviral drugs to affected households

Consider now transmission through a community of households. Suppose that every infection that an individual generates outside their own household is of a randomly selected community member. During the containment phase of the pandemic, the force of infection acting on a susceptible from outside the household is negligible relative to the force of infection exerted by an infectious household member. Using this we can show that, in a community of households, the number of doses of antiviral drugs dispensed per community member is
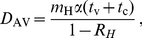
(5)where 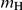 is the mean number of antiviral doses dispensed to the household of a newly-infected individual who is selected randomly from the community and 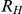 is the mean number of primary cases generated in the community by all the cases of a household outbreak initiated by a newly-infected individual who is selected randomly from the community. The derivation of (5) is outlined in Appendix S1. Equation (5) is the equivalent of (4) for a community of households. To be valid it requires, similarly to (4), that the reproduction number for infected households (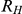) is less than 1.

Equation (5) can accommodate a variety of strategies for dispensing antiviral doses to households, including “every household member” and “every household case upon onset of their symptoms”. From (5) we conclude, as for (4), that an attempt to contain transmission uses relatively few doses of antiviral drugs, be it sustained containment of transmission or an attempt to contain transmission that is abandoned once it becomes clear that containment is not feasible.

The key to containing transmission in a community of households lies in the ability to bring 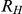 below 1. We now take a closer look at what is required to bring 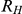 below 1, under the assumption that the effectiveness of antiviral drugs for reducing susceptibility and infectivity for the emerged virus strain is as estimated for currently circulating influenza strains. The effect of antivirals on reducing transmission is modeled as in Glass and Becker [25].

**Figure 4:**
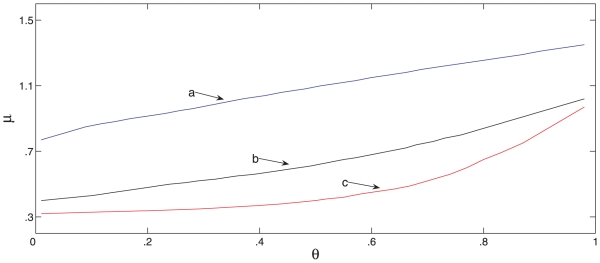
Curves for which the reproduction number for household outbreaks (*R_H_*) equals 1. The three 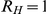 curves correspond to (a) antivirals are dispensed at onset of symptoms in the primary household case, (b) antivirals are dispensed two days after symptom onset in the primary case, and (c) no antiviral drugs are dispensed. For each of these three intervention scenarios, 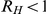 for every parameter point 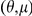 that lies below the 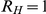 curve and 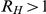 when 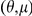 lies above the 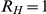 curve.

To show the roles of within and between household transmission we display results in terms of 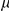, the mean number of individuals an infective infects outside their household, and 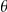, the probability that a susceptible household partner avoids infectious contact with a household case during the latter's infectious period. These interpretations of 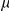 and 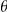 apply for a totally susceptible community in which antiviral drugs are not used. The curves in Figure 4 display values of 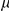 and 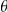 for which 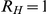 in three scenarios, namely (a) antivirals are dispensed at onset of symptoms in the primary case (two days after infection), (b) antivirals are dispensed two days after onset of symptoms in the primary household case, [2], [4] and (c) no antiviral drugs are dispensed. For each curve in Figure 4, parameter pairs 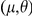 that lie below the curve satisfy 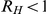, while 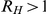 for parameter coordinates above the curve. By comparing the two lowest curves we see that dispensing drugs to all family members two days after onset of symptoms in the primary case expands the set of parameter values for which 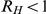 only a little. In contrast, dispensing drugs at onset of symptoms in the primary case expands the set of parameter values for which 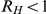 substantially. In other words, the set of scenarios for which containment becomes feasible is much larger when antiviral drugs are dispensed as soon as possible. When we compute values of 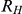 for parameter pairs 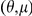 lying on curve (a) but assuming that no antiviral drugs are dispensed we obtain values in the range 1.9–2.7. This shows that antiviral drugs can bring a reproduction number that is well above 1 down to a value of 1 if they are dispensed at onset of symptoms in the primary case.

Timely dispensing of antiviral drugs is so important because, as reflected in the model, individuals infected with influenza become infectious prior to onset of symptoms and the bulk of their total infection potential has passed 2–3 days after symptom onset.

### Failure to present

Figure 4 illustrates that failure to present early can reduce the effectiveness of using antiviral drugs to mitigate transmission. People might fail to present because their clinical symptoms are not severe or present late due to delayed access to health services. It seems likely that use of antiviral drugs to contain transmission of pandemic H1N1 influenza in 2009 was not successful because the fraction of infected individuals who failed to present, or presented late, was too high. It is useful to have a way of determining how the fraction of primary household cases who do not present, or present late, limits the chance of containing transmission.

Let 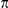 denote the proportion of primary household cases that fail to present early. Under the assumption that every member of a household whose primary case presents early receives a dose of antiviral drugs and that other households get no antiviral drugs the reproduction number for infected households becomes 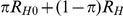, where 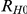 is the household reproduction number when no antiviral drugs are dispensed and 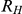 is the household reproduction number when antiviral drugs are dispensed to all infected households. This reproduction number is equal to 1 when
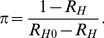
(6)Therefore, even when 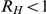, it is not possible to contain transmission if the proportion primary household cases who fail to present early is greater than the right hand side of (6).

**Figure 5:**
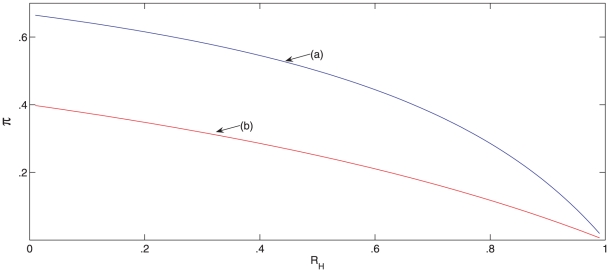
How the possibility to contain transmission depends on the proportion who fail to present early. Transmission can be contained for values of 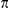 below the curve, where 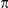 is the proportion of primary household cases who fail to present early, 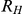 is the household reproduction number when all infected households receive antiviral drugs and the household reproduction number without antiviral drugs is 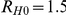 for curve (a) and 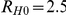 for curve (b).

Figure 5 shows the curves (6) for the values 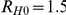 and 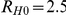 for various values of 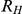 that might be obtained when antiviral drugs are dispensed to members of those households where the primary case presents early. For values of 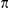 below the curve it is possible to contain transmission, but for values of 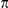 above the curve it is not. Suppose we can reduce the reproduction number for household outbreaks to 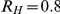 when antiviral drugs are dispensed to all infected households. Then containment of transmission requires that less than 29% of primary cases fail to present early when 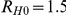, and less than 12% when 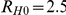.

### Are antiviral drugs effective against the newly emerged virus strain?

The motivation to create a stockpile of antiviral drugs is based on their demonstrated effectiveness against currently circulating influenza strains. There is no guarantee that these drugs will be equally effective, or even effective at all, against a newly emerged pandemic strain of influenza. Informed decisions about the use of antiviral drugs in a pandemic require effectiveness for reducing transmission to be established from incidence data collected early in a pandemic. In preparation we need to know what data, and how much, are required to establish effectiveness. Glass and Becker [25] consider this question by estimating two specific parameters, one quantifying the effect on susceptibility and the other the effect on infectivity. Here we look at establishing effectiveness by comparing mean outbreak size in households that receive antiviral drugs early and households that receive them late. We have to allow for different household sizes. Households of size one provide no information for our comparison and we restrict attention to households of sizes two, three and four. The Australian census data indicate that the relative frequency of households of size 2, 3 and 4 is about 50%, 25% and 25%. Allowing for size-biased sampling we expect to observe roughly an equal number of outbreaks in households of size 2, 3 and 4. Accordingly, we assume that we observe 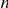 outbreaks in households that receive antiviral drugs at onset of symptoms in the primary household case in households of size 2, 3 and 4, making 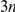 households. In addition, we assume that we observe 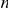 outbreaks in households that receive antiviral drugs late (two days after the onset of symptoms in the primary case) in households of size 2, 3 and 4, giving observations on another 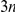 household outbreaks.

An Alexander-Govern test statistic [28] is computed for the comparison in households of a given size and values of these three test statistics are then summed and the null hypothesis of no effect is rejected if the sum exceeds the 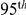 percentile of the 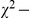
distribution with three degrees of freedom.

**Figure 6:**
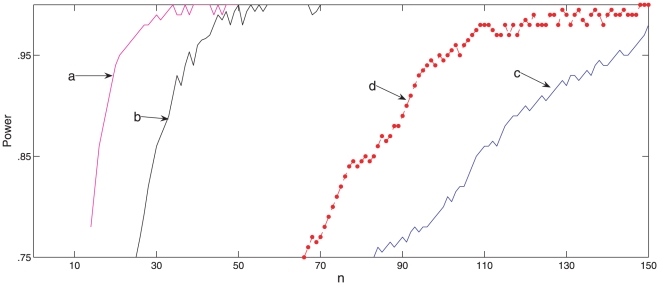
Power of a test to determine whether antiviral drugs are effective. Power of a test to compare the mean outbreak size in 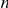 households who receive antiviral drugs early and 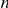 households who do not, for each of the household sizes 2, 3 and 4 (i.e. observations on 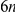 household outbreaks). A modified version of the Alexander-Govern test is used and power is estimated by applying the test to each of 500 data sets for each 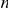 and each effect scenario. In curves (a), (b) and (c) the simulations assume that antiviral drugs partially reduce infectivity and their effect on susceptibility is to induce full, partial and zero protection, respectively. Simulations for curve (d) assume no effect on infectivity and a partial effect on susceptibility.

The power curve corresponding to a given antiviral effect scenario was estimated by simulating 500 data sets, applying the test to each data set and noting the fraction that reject the hypothesis of equal mean outbreak sizes. Figure 6 shows the estimated power curves for four antiviral effect scenarios similar to the ones considered by Glass and Becker [25], which enables a comparison of results and illustrates the findings. These scenarios are motivated by data on antiviral effects for currently circulating influenza strains, [25]. In the simulations we used 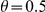 for the probability that an individual avoids being infected by a given household infective, in the absence of antiviral drugs.

The antiviral effect on susceptibility is to reduce the probability of transmission of an infection occurring during a contact by a factor 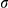 for a susceptible who is on antiviral drugs at the time. The effect on infectivity depends on the time when the infective starts taking the antiviral drug and is measured by the factor by which the area under the infectiousness function is reduced (i.e. the potential to infect others is reduced). Let 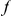 denote the factor by which the area under the infectiousness function is reduced when the individual commences taking the drug at onset of symptoms. The curves (a), (b) and (c) of Figure 6 show the power as 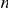 varies for the effect scenario with 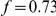 and 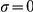, 0.5 and 1, respectively. Each point on the curve is obtained by simulating 500 data sets and observing the fraction that reject the no-effect hypothesis when our modified Alexander-Govern test is used. Curve (d) assumes the effect scenario with 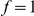, no effect on infectivity, and 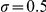, partial effect on susceptibility.

When considering the results in Figure 6 it is useful to keep in mind that monitoring infected households for cases is labour-intensive. In practice, monitoring more than 300 household outbreaks during the busy early stages of a pandemic would be very challenging. We would therefore like 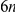, the total number of household outbreaks monitored, to be less than 300. Let us take a power of 80% as a minimum requirement. Inspecting curves (a), (b) and (c), which are generated by including a common effect on infectivity, illustrates that observations on 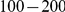 household outbreaks has a power of at least 80% of detecting an antiviral effect if there is also a moderate effect on susceptibility, but many more households are needed if susceptibility is not reduced. Comparing curves (b) and (d), which are generated by including a moderate effect on susceptibility, illustrates that a total of 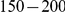 household outbreaks are needed to detect an effect if there is also a moderate effect on infectivity, but many more households are needed if the effect on infectivity is weak.

The hope that a direct comparison of mean outbreak size for households would require less data than a comparison based on specific parameters, as in [25], was not realised. The two approaches indicate approximately the same data needs. However, it is reassuring that a simple test based on minimal assumptions about the nature of transmission in the community can detect an antiviral effect with about the same amount of data.

## Discussion

Our aim was to see whether antiviral drugs should be used to mitigate general transmission following emergence of a future pandemic influenza strain. The main conclusion is a strong recommendation that liberal and timely use of antiviral drugs should be part of an attempt at local containment of transmission. The case for this lies in the substantial benefits of successful containment and the fact that the accumulated use of antiviral drugs over a period of successful containment is modest, even when the immigration rate of infected arrivals is high. The recommended plan for the attempt at containment must include abandoning prophylactic use of antiviral drugs once there is strong empirical evidence that containment is unlikely to succeed, because continued use of antiviral drugs to mitigate transmission when early containment fails is likely to use a very sizable supply of antiviral drugs with little benefit. The likelihood of successful containment should be evident by the time 350 cases have been reported and we have data on 200 household outbreaks.

Our basic first model acknowledges that the first few doses of antivirals dispensed per case can be targeted more effectively than a similar number of additional doses. That is, dispensing very many doses per cases is wasteful and likely to attract justified criticisms and objections. A practical way to dispense doses to close contacts only is to target household members of cases who present. Accordingly, our second model considers transmission through a community with a household structure and we considered delays in presentation. The conclusion that an attempt to contain transmission uses relatively few antiviral doses continues to hold in this setting. We also conclude that successful containment of transmission, if possible, requires early presentation by the primary household case. We cannot wait for laboratory confirmation of a strain-specific infection. The number of doses used by an attempt to contain transmission is so small that one can afford, while a chance of containing transmission remains, to be liberal in dispensing doses to household members of any early presenter with clinical symptoms that are consistent with a pandemic-strain infection.

Next we allowed for failure by a fraction of primary household cases to present soon after onset of symptoms. The conclusion is that, even when antivirals are adequately effective, containment is not possible if more than a modest fraction of primary household cases fail to present early. In our illustration we required the proportion of primary cases who fail to present early to be smaller than 20%. This 20% includes asymptomatic cases, mildly-symptomatic cases who do not bother to present and symptomatic cases unable to gain timely access to a health service provider.

Finally, appropriate data must be collected at the start of the local outbreak to estimate the initial reproduction number and to confirm that antiviral drugs do reduce transmission of the new strain of influenza virus. It is concluded that we can expect to detect an antiviral effect on transmission from data on 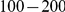 household outbreaks only if the antiviral drug has a moderate effect on both susceptibility and infectivity (of the same order as for currently circulating strains of influenza).

These conclusions are not consequences of the simplifying assumptions made in the specific models of this paper. They rely primarily on the threshold result that 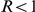 implies containment and this result holds under a very wide range of community settings and disease characteristics. The likelihood of achieving 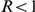 depends critically on the transmission characteristics of the newly emerged disease and our ability to deliver antiviral drugs early enough to affected households. With pandemic H1N1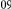 we were close in some locations. For example, in Western Australia, as in some other localities, most early cases were imported infections indicating that 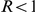 was maintained for a substantial period, [31]. With clear understanding and confidence that continued liberal use of antiviral drugs is the best option at that stage it may have been possible to sustain 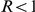 longer.

## Supporting Information

Appendix S1 **Outline of the derivation of results for transmission in a community of households.**
(DOC)
