# Supplementary material for: Can Antiviral Drugs Contain Pandemic Influenza Transmission?
Source: PLoS One. 2011 Mar 28;6(3):e17764. doi: 10.1371/journal.pone.0017764 (PMC3065466; doi:10.1371/journal.pone.0017764)
Supplement: Appendix S1 — Outline of the derivation of results for transmission in a community of households. (DOC) [file pone.0017764.s001.doc]

**Appendix S1. Outline of the derivation of results for transmission in a community of households**

The probability that a randomly selected community member belongs to a household of size $j$ is given by , where is the proportion of households of size in the community. Let $c=(c_{1},c_{2},c_{3},\ldots)$ denote the chain of infection in a household outbreak, where $c_{j}$ is the number of cases in generation $j$. More specifically, $c_{1}=1$ corresponds to the primary case, $c_{2}$ is the number of cases infected by the primary case and similarly, for any $j\geq2$, $c_{j}$ gives the number of cases resulting from contacts with cases of the previous generation. Let $p(c|j)$ denote the probability that transmission chain $c$ results in a household of size $j$ when the outbreak is initiated by a single primary case and evolves independently of external sources of infection. Let $m(c,j)$ denote the number of doses of antiviral drugs dispensed for such a household outbreak. Note that $m(c,j)=j$ if one dose is dispensed to each household member and if only cases receive a dose. The mean number of antiviral doses dispensed to the household of a newly-infected individual who is selected randomly from the community is

$$m_{\text{H}}=\sum_{j} g_{j}\sum_{c} m(c,j)p(c|j).$$

Let $\mu_{j}$ denote the mean number of individuals a $j^{\text{th}}$ generation household case infects in the community. We allow this mean to depend on the generation because the effect of antivirals on the infectivity of an individual depends on the time they are infected relative to the time when antiviral drugs are dispensed to household members. Infection of a randomly selected community member results in a household chain of transmission **c** with probability $p(c)=\sum_{j} g_{j}p(c|j)$. Using the idea that each newly-infected person, if selected randomly from the community, generates a new independent transmission process with the same eventual mean number of cases, we find the mean total number of eventual cases to be

$N_{\text{c}}=\frac{\nu_{\text{H}}}{1-R_{H}},$ (7)

where $\nu_{\text{H}}=\sum_{c} p(c)\sum_{j} c_{j}$ is the mean size of a random household outbreak and $R_{H}=\sum_{c} p(c)\sum_{j} c_{j}\mu_{j}$ is the mean number of primary cases generated in the community by all the cases of a random household outbreak. These expressions assume that the primary household case is an individual chosen at random from the community and (7) requires that $R_{H}$, the reproduction number for infected households, be less than 1.

A similar argument gives expression (5) for the mean number of doses of antiviral drugs dispensed per community member, which also requires that $R_{H}<1$.

We say that transmission is contained when we can keep $R_{H}$ below 1, because this assures a small eventual attack rate. For a better understanding of the requirement that $R_{H}<1$ when the focus of intervention is on household outbreaks we need to look at $p(c)$. Assume that every household member receives a dose of antiviral drugs when the primary household case presents, so that $m(c,j)=j$. We use the Reed-Frost model, see [26, 27], to describe the within-household transmission. In the absence of interventions, the probability distribution for the size of the household outbreak is expressed in terms of the parameter $\theta$, the probability that a susceptible household member avoids infection by a single household infective over the duration of the latter's infectious period. We may write $\theta=\exp(-\int_{0}^{\infty} \lambda_{x} dx)$, where $\lambda_{x}$ is the infectiousness function (Becker [27], pp 45-47). For the effect of antiviral drugs on susceptibility and infectivity of individuals we follow the modeling of Glass and Becker [25]. In brief, reduction in susceptibility is reflected by reducing the per contact probability of transmission by a constant factor, while the susceptible is on antiviral drugs. This has the effect of changing $\theta$ to $\theta^{\sigma}$. Reduction in infectivity is described via a model for the viral load carried by a case. In the absence of antiviral drugs the viral load is described by a deterministic birth process with a constant rate from the time of infection until time 2 (days), when a death rate is added to reflect the response of the immune system. Reduction in infectivity as a result of taking antiviral drugs is reflected by adding, to the virus population dynamics, a constant death rate (equivalently subtracting a constant from the birth rate) while the individual is on antiviral drugs. The parameter values of the model, as in Glass and Becker [25], are chosen to be consistent with observed data.
